# 4212 An age-dependent, rescuable defect in intestinal barrier repair is associated with an immature enteric glial network in a neonatal pig model of intestinal ischemia

**DOI:** 10.1017/cts.2020.290

**Published:** 2020-07-29

**Authors:** Amanda Ziegler, Anastasia E. Sheridan, Tiffany A. Pridgen, Jack Odle, Laurianne Van Landeghem, Anthony T. Blikslager

**Affiliations:** 1 North Carolina State University

## Abstract

OBJECTIVES/GOALS: An age-dependent restitution defect in our neonatal pig intestinal ischemia model is rescued by unknown factors within homogenized mucosa of weaned pigs. A postnatally maturing network of enteric glia regulates the epithelial barrier, so we aim to show rescue is due to replacement of glial factors. METHODS/STUDY POPULATION: Jejunal tissues from suckling or weaned pigs were assessed by RNAseq and processed for immunofluorescent histology and 3-D volume imaging. Jejunal ischemia was surgically induced in weaned pigs and injured mucosa was recovered ex vivo with or without the glial inhibitor fluoroacetate (FA) while monitoring transepithelial electrical resistance (TER). RESULTS/ANTICIPATED RESULTS: Ingenuity Pathways Analysis of RNAseq data revealed significant suppression of numerous pathways critical for epithelial wound healing in suckling pigs (Z-score <−2 for of nine key pathways). Volume imaging studies confirmed lower density (P≤0.05) and complexity of the subepithelial glial network in suckling pigs. Treatment with FA inhibited TER recovery (P<0.0001) and restitution (P<0.05) in weaned pigs, mimicking the suckling pig phenotype and supporting glia as an important regulator of restitution in our model. DISCUSSION/SIGNIFICANCE OF IMPACT: These findings provide important evidence that a developing glial network may be critical to the postnatal development of intestinal barrier repair mechanisms. Ongoing work will explore glial-epithelial interactions *in vitro* to further define postnatal development of barrier repair.

